# Supplementary material for: Reversing sintering effect of Ni particles on γ-Mo2N via strong metal support interaction
Source: Nat Commun. 2021 Nov 30;12:6978. doi: 10.1038/s41467-021-27116-8 (PMC8632928; doi:10.1038/s41467-021-27116-8)
Supplement: Supplementary file 1 — Supplementary Information [file 41467_2021_27116_MOESM1_ESM.pdf]

Supplementary Information

Reversing sintering effect of Ni particles wetting on γ-Mo_2N support

Lili Lin[1,2#], Jinjia Liu[3,4#], Xi Liu[5*], Zirui Gao[2], Ning Rui[6], Siyu Yao[7], Feng Zhang[8], Chang Liu[9], Lili Han[6], Feng Yang[10], Sen Zhang[9], Xiao-dong Wen[3,4], Sanjaya D. Senanayake[6], Jose A. Rodriguez[6,8*], Ding Ma[2*]

1. Institute of Industrial Catalysis, State Key Laboratory of Green Chemistry Synthesis Technology, College of Chemical Engineering, Zhejiang University of Technology, Hangzhou 310014, Zhejiang, P. R. China
2. Beijing National Laboratory for Molecular Sciences, College of Chemistry and Molecular Engineering and College of Engineering and BIC-ESAT Peking University, Beijing 100871, P. R. China
3. State Key Laboratory of Coal Conversion, Institute of Coal Chemistry, Chinese Academy of Sciences, Taiyuan, China
4. National Energy Centre for Coal to Liquids, Synfuels China Co. Ltd, Beijing, China
5. School of Chemistry and Chemical Engineering, In-situ Center for Physical Science, Shanghai Jiao Tong University, Shanghai, China
6. Chemistry Division, Brookhaven National Laboratory, Upton, New York 11973, USA
7. Key Laboratory of Biomass Chemical Engineering of Ministry of Education, College of Chemical and Biological Engineering, Zhejiang University, Hangzhou, 310027 China
8. Materials Science and Chemical Engineering Department, State University of New York, Stony Brook, New York, US
9. Department of Chemistry, University of Virginia, Charlottesville, Virginia 22904, United States
10. Department of Chemistry, Southern University of Science and Technology, Shenzhen 518055, China

Supplementary Figures and Tables

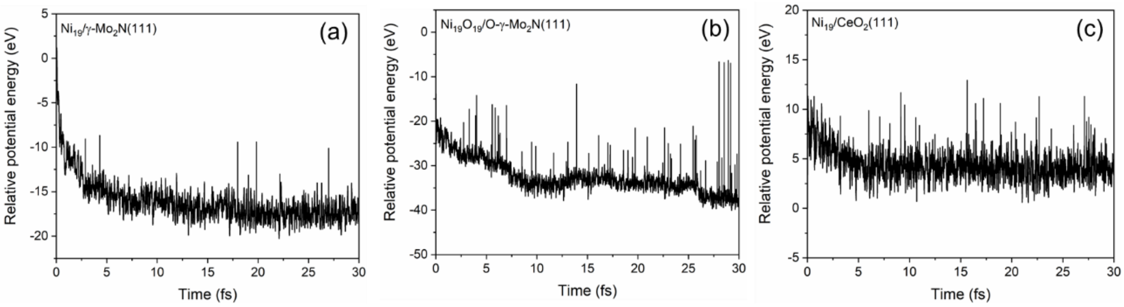

Supplementary Figure 1. Potential energies of (a) Ni_{19}/γ-Mo_2N(111), (b) $Ni_{19}O_{19}$/O-γ-Mo_2N(111) and (c) Ni_{19}/CeO_2(111) during AIMD simulations.

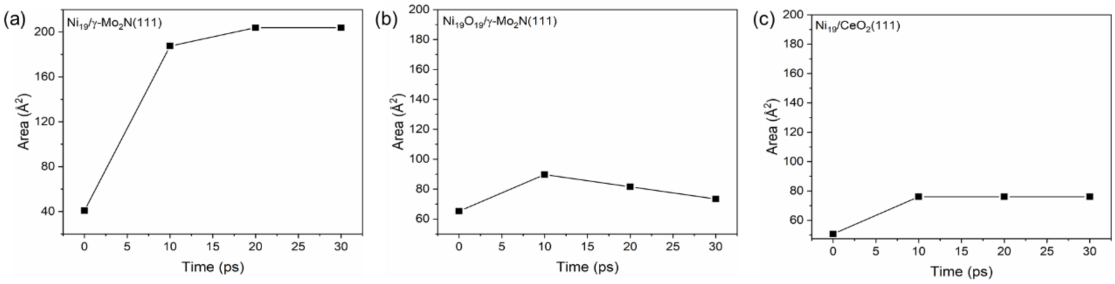

Supplementary Figure 2. The interface area with function of time for (a) Ni_{19}/γ-Mo_2N(111), (b) $Ni_{19}O_{19}$/γ-Mo_2N(111) and (c) Ni_{19}/CeO_2(111).

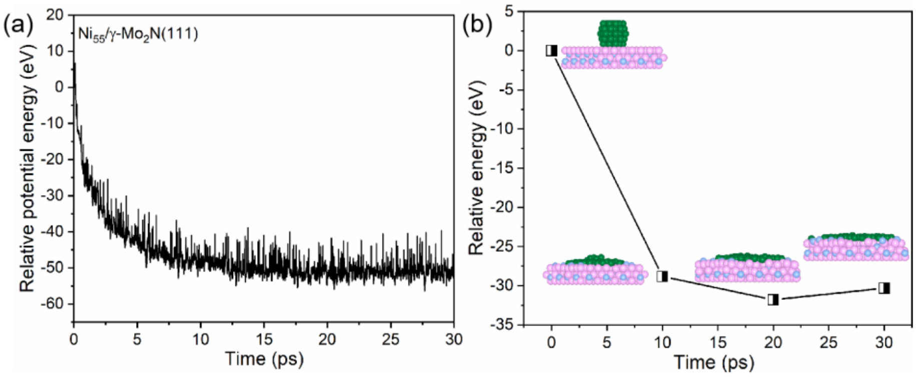

Supplementary Figure 3. (a) Potential energy of Ni_{55}/γ-Mo_2N(111) during AIMD simulation. (b) The relative energy change of Ni_{55}/γ-Mo_2N(111) from static DFT calculations.

Supplementary Table 1. The Ni K edge EXAFS fitting results of Ni-4nm/γ-Mo_2N and Ni-4nm/CeO_2 catalysts

Sample	Temp. (ºC)	Shell	Bond length (Å)	Coordination Number	σ^2 (Å)	E_0 shift (eV)
Ni-4nm/γ-Mo_2N	400	Ni-Ni	2.49±0.01	4.5±0.6	0.01	-6.9
		Ni-N	1.9±0.02	1.5±0.6	0.003	
	590	Ni-Ni	2.49±0.01	2.6±1.0	0.004	-8.3
		Ni-Mo	2.62±0.03	5.4±1.1	0.016	
		Ni-N	2.00±0.03	1.7±0.7	0.025	
Ni-4nm/CeO_2	590	Ni-Ni	2.49±0.01	10.8±1.2	0.006	0.2

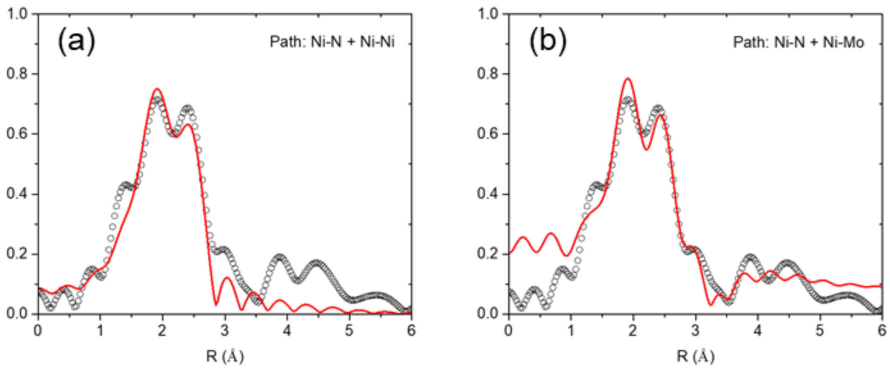

Supplementary Figure 4. The detailed description of EXAFS Ni K edge fitting results of Ni-4nm/γ-Mo_2N-400. (a) Ni-N + Ni-Ni paths, (b) Ni-N + Ni-Mo paths. The latter one cannot get reasonable fitting results and good fitting quality.

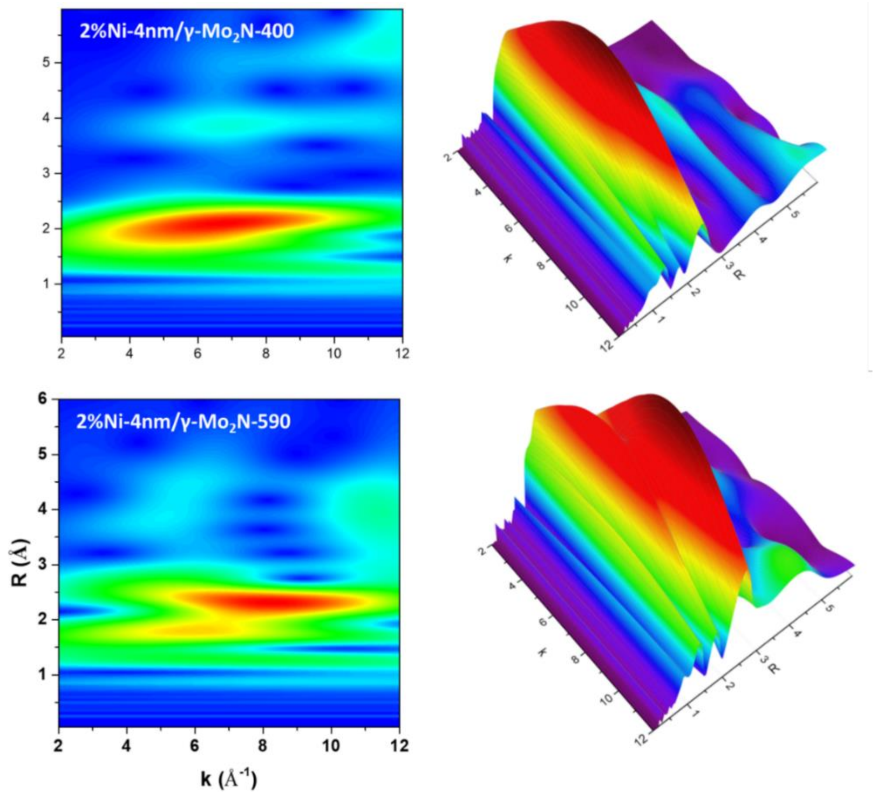

Supplementary Figure 5. The wavelet transformation (WT) of the EXAFS spectra of the 2% Ni-4nm catalyst at 400 and 590 °C under reduction condition.

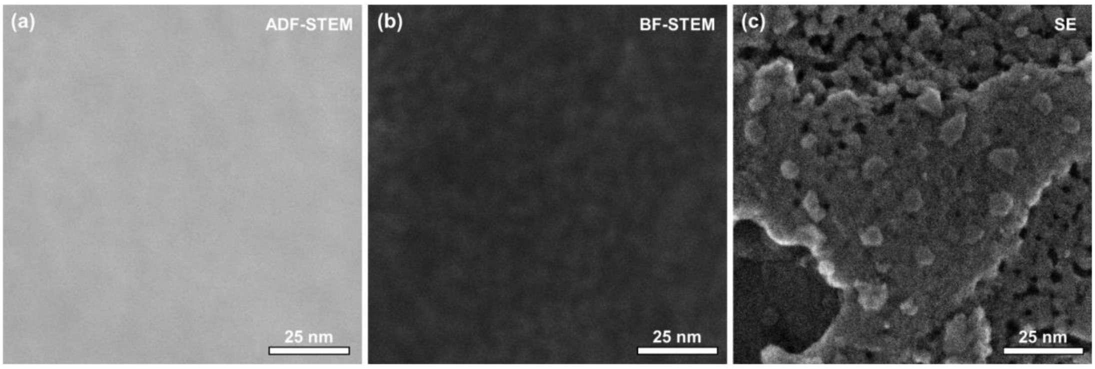

Supplementary Figure 6. The STEM images of (a) ADF, (b) BF and (c) SE.

Ni_3N

ref no.	h	k	l	d [A]	c
[1]	-1	1	0	4.0031	
[2]	1	-1	0	4.0031	
[3]	0	1	0	4.0031	
[4]	1	0	0	4.0031	
[5]	0	-1	0	4.0031	
[6]	-1	0	0	4.0031	
[7]	0	-1	-1	2.9318	
[8]	0	1	1	2.9318	
[9]	1	-1	-1	2.9318	
[10]	-1	1	-1	2.9318	
[11]	-1	0	-1	2.9318	
[12]	1	-1	1	2.9318	
[13]	1	0	1	2.9318	
[14]	-1	1	1	2.9318	
[15]	0	-1	1	2.9318	
[16]	1	0	-1	2.9318	
[17]	0	1	-1	2.9318	
[18]	-1	0	1	2.9318	
[19]	-2	1	0	2.3112	
[20]	1	-2	0	2.3112	
[21]	2	-1	0	2.3112	
[22]	-1	2	0	2.3112	

Ni_4N

ref no.	h	k	l	d [A]	c
[1]	1	0	0	3.7151	
[2]	0	1	0	3.7151	
[3]	0	0	1	3.7151	
[4]	0	0	-1	3.7151	
[5]	0	-1	0	3.7151	
[6]	-1	0	0	3.7151	
[7]	0	-1	1	2.6270	
[8]	1	0	-1	2.6270	
[9]	1	0	1	2.6270	
[10]	-1	-1	0	2.6270	
[11]	1	1	0	2.6270	
[12]	-1	0	-1	2.6270	
[13]	-1	0	1	2.6270	
[14]	0	1	1	2.6270	
[15]	0	1	-1	2.6270	
[16]	-1	1	0	2.6270	
[17]	1	-1	0	2.6270	
[18]	0	-1	-1	2.6270	
[19]	-1	1	-1	2.1449	
[20]	1	-1	1	2.1449	
[21]	-1	-1	-1	2.1449	
[illegible]	[illegible]	[illegible]	[illegible]	[illegible]	

NiO

ref no.	h	k	l	d [A]
[1]	1	1	-1	2.4122
[2]	1	1	1	2.4122
[3]	-1	-1	1	2.4122
[4]	1	-1	-1	2.4122
[5]	-1	1	-1	2.4122
[6]	1	-1	1	2.4122
[7]	-1	-1	-1	2.4122
[8]	-1	1	1	2.4122
[9]	0	0	-2	2.0890
[10]	0	-2	0	2.0890
[11]	0	0	2	2.0890
[12]	2	0	0	2.0890
[13]	-2	0	0	2.0890
[14]	0	2	0	2.0890
[15]	-2	0	-2	1.4771
[16]	2	0	2	1.4771
[17]	0	-2	-2	1.4771
[18]	2	2	0	1.4771
[19]	0	2	2	1.4771
[20]	-2	-2	0	1.4771
[21]	-2	0	2	1.4771
[22]	2	-2	0	1.4771

Ni

ref no.	h	k	l	d [A]	c
[1]	-1	-1	-1	2.0367	
[2]	1	1	1	2.0367	
[3]	1	1	-1	2.0367	
[4]	1	-1	-1	2.0367	
[5]	-1	1	1	2.0367	
[6]	-1	-1	1	2.0367	
[7]	1	-1	1	2.0367	
[8]	-1	1	-1	2.0367	
[9]	0	0	-2	1.7638	
[10]	2	0	0	1.7638	
[11]	0	-2	0	1.7638	
[12]	-2	0	0	1.7638	
[13]	0	0	2	1.7638	
[14]	0	2	0	1.7638	
[15]	0	-2	2	1.2472	
[16]	2	-2	0	1.2472	
[17]	-2	2	0	1.2472	
[18]	2	0	2	1.2472	
[19]	-2	-2	0	1.2472	
[20]	2	2	0	1.2472	
[21]	0	-2	-2	1.2472	
[22]	-2	0	2	1.2472	

Mo_2N

ref no.	h	k	l	d [A]	c
[1]	0	1	1	3.7187	
[2]	0	1	-1	3.7187	
[3]	0	-1	1	3.7187	
[4]	1	0	-1	3.7187	
[5]	-1	0	1	3.7187	
[6]	1	0	1	3.7187	
[7]	0	-1	-1	3.7187	
[8]	-1	0	-1	3.7187	
[9]	1	1	2	2.3845	
[10]	-1	-1	-2	2.3845	
[11]	-1	1	-2	2.3845	
[12]	1	1	-2	2.3845	
[13]	1	-1	2	2.3845	
[14]	1	-1	-2	2.3845	
[15]	-1	1	2	2.3845	
[16]	-1	-1	2	2.3845	
[17]	1	0	3	2.2512	
[18]	0	-1	-3	2.2512	
[19]	-1	0	-3	2.2512	
[20]	0	1	3	2.2512	
[21]	0	1	-3	2.2512	

MoN

ref no.	h	k	l	d [A]	c
[1]	0	0	-1	5.5200	
[2]	0	0	1	5.5200	
[3]	0	-1	0	4.9060	
[4]	-1	0	0	4.9060	
[5]	-1	1	0	4.9060	
[6]	0	1	0	4.9060	
[7]	1	-1	0	4.9060	
[8]	1	0	0	4.9060	
[9]	1	-1	-1	3.6670	
[10]	1	-1	1	3.6670	
[11]	-1	1	-1	3.6670	
[12]	-1	1	1	3.6670	
[13]	0	-1	1	3.6670	
[14]	-1	0	1	3.6670	
[15]	-1	0	-1	3.6670	
[16]	1	0	-1	3.6670	
[17]	1	0	1	3.6670	
[18]	0	-1	-1	3.6670	
[19]	0	1	-1	3.6670	
[20]	0	1	1	3.6670	
[21]	-1	2	0	2.8325	
[22]	1	-2	0	2.8325	
[23]	-2	1	0	2.8325	
[24]	2	-1	0	2.8325	

Mo

ref no.	h	k	l	d [A]	d
[1]	0	-1	-1	2.2255	
[2]	-1	0	-1	2.2255	
[3]	1	0	1	2.2255	
[4]	1	1	0	2.2255	
[5]	-1	-1	0	2.2255	
[6]	0	1	1	2.2255	
[7]	1	0	-1	2.2255	
[8]	0	1	-1	2.2255	
[9]	-1	1	0	2.2255	
[10]	1	-1	0	2.2255	
[11]	-1	0	1	2.2255	
[12]	0	-1	1	2.2255	
[13]	0	0	2	1.5737	
[14]	0	-2	0	1.5737	
[15]	2	0	0	1.5737	
[16]	0	2	0	1.5737	
[17]	0	0	-2	1.5737	
[18]	-2	0	0	1.5737	
[19]	-1	2	1	1.2849	
[20]	1	1	2	1.2849	
[21]	-1	2	-1	1.2849	

Supplementary Figure 7. d spacing of 2.6Å analysis and comparison according to all possible phases in Ni/γ-Mo_2N catalyst.

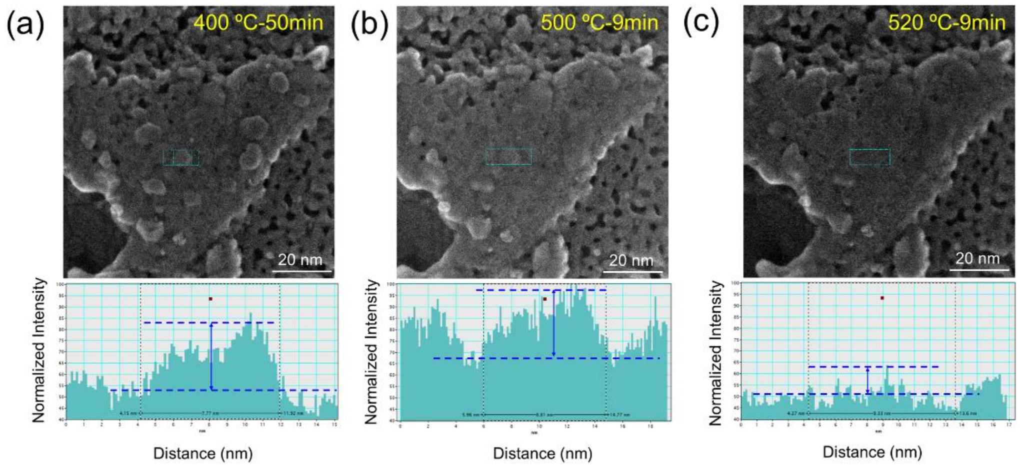

Supplementary Figure 8. The normalized secondary electron intensity of Ni species at (a) 400 °C, (b) 500 °C and (c) 520 °C. The intensity of Ni species at 520 °C sample was much weaker than those of the 400 and 500 °C samples, which is another evidence that the Ni particles has reverse sintering at high temperature.

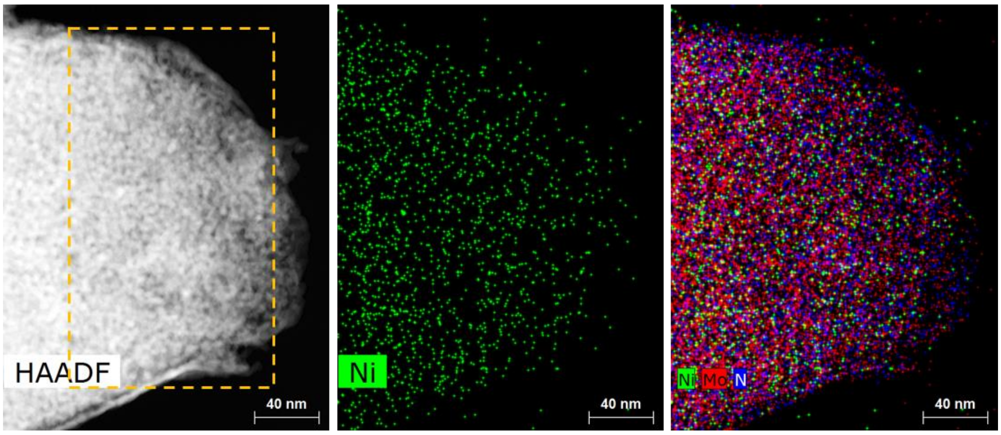

Supplementary Figure 9. STEM-HAADF image and EDS mapping of ex-situ sample of Ni-4nm/γ-Mo_2N-590 catalyst.

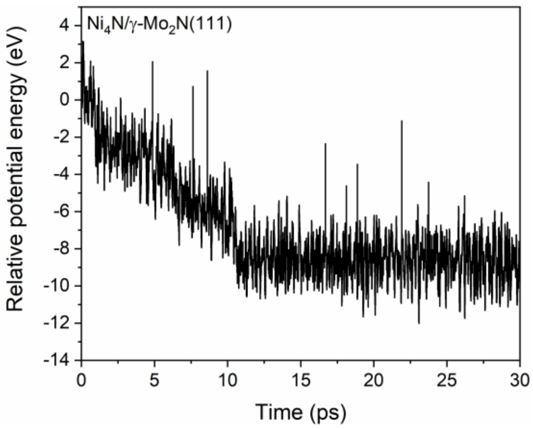

Supplementary Figure 10. Relative potential energies of $Ni_4N/\gamma\text{-}Mo_2N(111)$ during AIMD simulations.

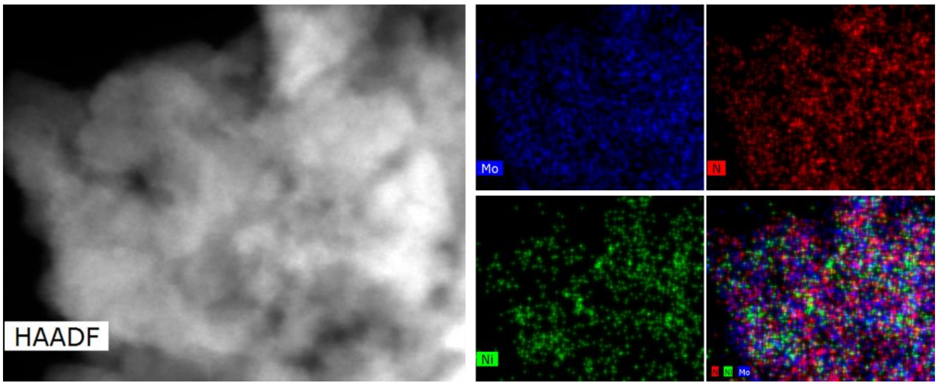

Supplementary Figure 11. STEM-HAADF image and EDS mapping of Ni/γ-Mo_2N after long term CO_2 hydrogenation reaction.

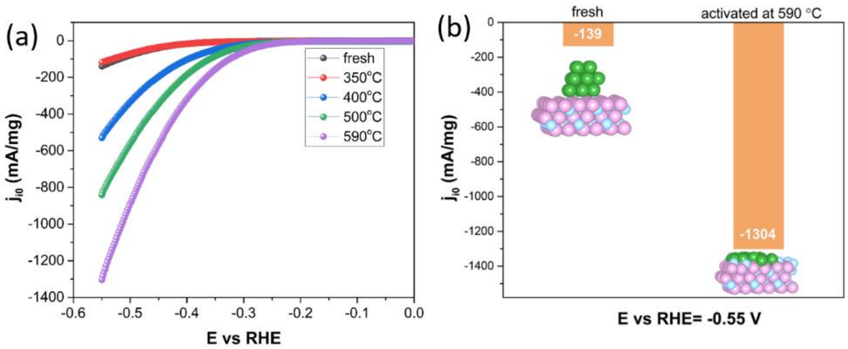

Supplementary Figure 12. The performance evaluation for Ni-4nm/γ-Mo_2N treated at different temperature. (a) (b) the activity of hydrogen evaluation reaction over Ni-4nm/γ-Mo_2N catalyst at the state of fresh, activated at 350, 400, 500 and 590 ºC. The activated catalysts were passivated by CO_2 before the HER performance test.

It has also been demonstrated the reverse sintering effect in the Ni/γ-Mo_2N catalyst is able to enhance the water splitting activity by increasing the exposure of the active metal sites. Using the electrocatalytic hydrogen evolution reaction (HER) as the model reaction, we have evaluated the HER activity of the Ni-4nm/γ-Mo_2N catalysts activated at different temperature in the reductive atmosphere. Based on Figure 5f and g, it is clear that with the spread of the Ni atoms, the specific current density has been enhanced significantly. Comparing the fresh catalyst with the Ni-4nm/γ-Mo_2N-590 catalyst, the hydrogen evolution rate increased by about 10 times. Therefore, the reverse sintering effect can be used to maximize the dispersion and utilization efficiency of metals using simple treatments.
