# Supplementary material for: Reversing sintering effect of Ni particles on γ-Mo2N via strong metal support interaction
Source: Nat Commun. 2021 Nov 30;12:6978. doi: 10.1038/s41467-021-27116-8 (PMC8632928; doi:10.1038/s41467-021-27116-8)
Supplement: Supplementary file 3 — Description of Additional Supplementary Files [file 41467_2021_27116_MOESM3_ESM.pdf]

Description of Additional Supplementary Files

File Name: Supplementary Movie 1
Description: The MD simulation of Ni19 on γ-Mo2N for 30 ps (side view)

File Name: Supplementary Movie 2
Description: The MD simulation of Ni19 on γ-Mo2N for 30 ps (top view)

File Name: Supplementary Movie 3
Description: The MD simulation of Ni19O19 on γ-Mo2N for 30 ps (side view)

File Name: Supplementary Movie 4
Description: The MD simulation of Ni19O19 on γ-Mo2N for 30 ps (top view)

File Name: Supplementary Movie 5
Description: The MD simulation of Ni19 on CeO2 for 30 ps (side view)

File Name: Supplementary Movie 6
Description: The MD simulation of Ni19 on CeO2 for 30 ps (top view)

File Name: Supplementary Movie 7
Description: The MD simulation of Ni55 on γ-Mo2N for 30 ps (side view)

File Name: Supplementary Movie 8
Description: The MD simulation of Ni55 on γ-Mo2N for 30 ps (top view)

File Name: Supplementary Movie 9
Description: The record of environmental SE/STEM characterization of Ni-4nm/γ-Mo2N catalyst in the flow of H2/N2 at a ratio of 3:1 at 520 ºC.

File Name: Supplementary Movie 10
Description: The structure evolution of Ni20N5 on γ-Mo2N(111) in AIMD calculation for 30 ps (side view)

File Name: Supplementary Movie 11
Description: The structure evolution of Ni20N5 on γ-Mo2N(111) in AIMD calculation for 30 ps (top view)
